# Progression and natural history of Atypical Parkinsonism (ATPARK): Protocol for a longitudinal follow-up study from an underrepresented population

**DOI:** 10.1371/journal.pone.0325624

**Published:** 2025-06-26

**Authors:** Ravi Yadav, Saikat Dey, Ravichandiran Kumar, Athira P. Mohanan, Geethu T. Vasudevan, Manasi Harish, Nitish Kamble, Vikram V. Holla, Rohan R. Mahale, Pooja Mailankody, Monojit Debnath, Jitender Saini, Keshav Kumar, Anita Mahadevan, Sarada Subramanian, Phalguni Alladi, Indrani Datta, Binu V. Sreekumarannair, Priya Thomas, Anish Mehta, Albert Stezin, Madhura Ingalhalikar, Sweta Ramdas, Deepthi R. Bathula, Pramod Kumar Pal

**Affiliations:** 1 Department of Neurology, National Institute of Mental Health and Neurosciences, Bangalore, India; 2 Department of Human Genetics, National Institute of Mental Health and Neurosciences, Bangalore, India; 3 Department of Neuroimaging and Interventional Radiology, National Institute of Mental Health and Neurosciences, Bengaluru, Karnataka, India; 4 Department of Clinical Psychology, National Institute of Mental Health and Neurosciences (NIMHANS), Bangalore, Karnataka, India; 5 Department of Neuropathology, National Institute of Mental Health and Neurosciences (NIMHANS), Bangalore, Karnataka, India; 6 Department of Neurochemistry, National Institute of Mental Health and Neurosciences (NIMHANS), Bangalore, Karnataka, India; 7 Department of Clinical Psychopharmacology and Neurotoxicology, National Institute of Mental Health and Neurosciences (NIMHANS), Bangalore, Karnataka, India; 8 Department of Biophysics, National Institute of Mental Health and Neurosciences (NIMHANS), Bangalore, Karnataka, India; 9 Department of Biostatistics, National Institute of Mental Health and Neurosciences (NIMHANS), Bangalore, Karnataka, India; 10 Department of Psychiatric Social Work, National Institute of Mental Health and Neurosciences (NIMHANS), Bangalore, Karnataka, India; 11 Department of Neurology, Ramaiah Medical College and Hospitals, Bangalore, India; 12 Clinician Scientist, Indian institute of Science, Centre for brain research, Bangalore, India; 13 Symbiosis Center for Medical Image Analysis and Symbiosis Institute of Technology, Symbiosis International University, Pune, Maharashtra, India; 14 Indian Institute of Science, Bangalore, India; 15 Department of Computer Science and Engineering, Indian Institute of Technology Ropar, Bara Phool, Punjab, India; PLOS: Public Library of Science, UNITED KINGDOM OF GREAT BRITAIN AND NORTHERN IRELAND

## Abstract

**Background:**

Atypical Parkinsonian Syndromes (APS) form the third largest group of neurodegenerative disorders including Progressive Supranuclear Palsy (PSP), Multiple System Atrophy (MSA), and Corticobasal Syndrome (CBS). These conditions are characterized by rapid progression, poor prognosis, low survival rates, and limited treatment options. Few studies have suggested that genetic, environmental factors and inflammation contribute to the pathobiology of these complex disorders, however, the etiology of disease and progression remains unclear.

**Methods:**

A multicenter prospective longitudinal (3-time point) study will be conducted with a total sample size of 400 across all the groups (PSP, MSA, CBS). Patients with APS will be recruited after a detailed evaluation by movement disorder specialists and obtaining valid informed consent. The socio-demographic data and whole exome sequencing will be performed only at the baseline. Non-invasive procedures such as neurological and cognitive assessments, sleep quality assessments including polysomnography, brain imaging, and retinal imaging will be conducted at each time point. In addition, gene expressions, methylation patterns, inflammatory cytokines, disease-associated pathological proteins (Tau, pTau-181, α-synuclein and β-amyloid), non-targeted proteomics, skin biopsy, and iPSC will be performed at each time point eventually. The statistical analysis will be performed, followed by the developing of machine learning (ML) models.

**Expected outcomes and conclusion:**

This unique native dataset in APS will enhance our understanding of the molecular mechanisms driving pathological protein aggregation and disease progression. Furthermore, the longitudinal design of the study enables a detailed examination of symptom development, progression, and management. The ML models combined with advanced imaging techniques will aid in early diagnosis, differentiation among APS types, and the development of future clinical trials and treatment strategies.

## Introduction

Atypical Parkinsonian Syndromes (APS) also known as Parkinson-plus syndromes, refer to a heterogeneous group of neurodegenerative disorders that are distinct from Parkinson’s disease (PD) [1]. Progressive Supranuclear Palsy (PSP), Multiple System Atrophy (MSA), Corticobasal Syndrome (CBS), Lewy Body Dementia (LBD), Frontotemporal Dementia with Parkinsonism (FTD-P) and Normal Pressure Hydrocephalus (NPH) are the significant types. The cumulative prevalence of APS is 5% to 10% of PD populations [2]. The APS are often misdiagnosed as PD in the earlier stages [3]. However, some of the clinical features (red flags) that include early frequent falls, early cognitive impairment, more rapid progression, poor response to levodopa, severe autonomic dysfunction, and early bulbar symptoms such as dysphagia, dysphonia, and dysarthria are the key distinguishing factors for the diagnosis of APS from PD [1,4].

The pathological hallmarks of the APS are characterized by the aggregation of tau protein (PSP, CBS, FTD-P), and α-synuclein (MSA) in different brain regions [5,6]. Although most of the cases are sporadic, familial monogenic APS was also reported. Several genetic risk factors were identified through genome-wide association and candidate gene association studies. Most of these were accumulated in *MAPT*, *STX6*, *EIF2AK3*, *MOBP*, *RUNX2*, *SLCO1A2*, *TRIM11*, *LRRK2*, *PGRN*, *C9ORF72*, *FUS*, *DCTN1*, *COQ2*, *SNCA,* and other genes [7,8]. Furthermore, a few studies have suggested the influence of various environmental factors, such as prolongedborewell water consumption, exposure to agricultural pesticides, living in areas near industrial waste, and lower educational attainment on the risk of PSP [9–11]. Increased methylation at the promoter regions of *DLX1*, *ARL17A*, *ARL17B,* and *MOBP* genes was also observed in APS [12–14]. Moreover, recent reports identified several immune players dysregulated in the brain milieu of tauopathies and synucleinopathies [15,16]. Importantly, several proinflammatory cytokines such as TNF-α, IL-1β, IL-2, IL-4, IL-6 etc. along with microglial activation have been shown to play important roles in APS [17–19]. These were also evident in magnetic resonance imaging (MRI) and positron emission tomography (PET) which indicate the hegemony of pro-inflammatory cytokines in the central nervous system [20,21]. This led to neurodegeneration mainly in the midbrain and brainstem regions depending on the pathology which are involved in controlling various cognitive functions [22,23]. On the other hand, retinal changes over time were predicted as a valuable biomarker for monitoring the progression of APS [24]. Despite these advances, the mechanism underlying disease onset and progression has not been clearly understood.

A definite diagnosis for APS is only possible through autopsy, making early diagnosis a challenge for healthcare professionals. Predicting the progression and survival of patients with APS remains challenging due to the lack of reliable laboratory tests or neuroimaging markers. The heterogeneity of these syndromes further complicates efforts to develop effective therapies. As symptoms of APS progress rapidly, it is essential to provide preventive care for both patients and caregivers. There are very few longitudinal cohort studies globally, hardly any from Asia and none from India. Therefore, in the current study protocol, we aim to identify clinical, plasma, cerebrospinal fluid (CSF), genetic, epigenetic, sleep, eye movement, and brain imaging biomarkers in APS by serial recording and follow-up at least three time points within a five-year timeframe which will further help us to understand the neurodegeneration and functional disintegration of biological functions with time in APS.

## Methods

### Overview

**Fig 1:**
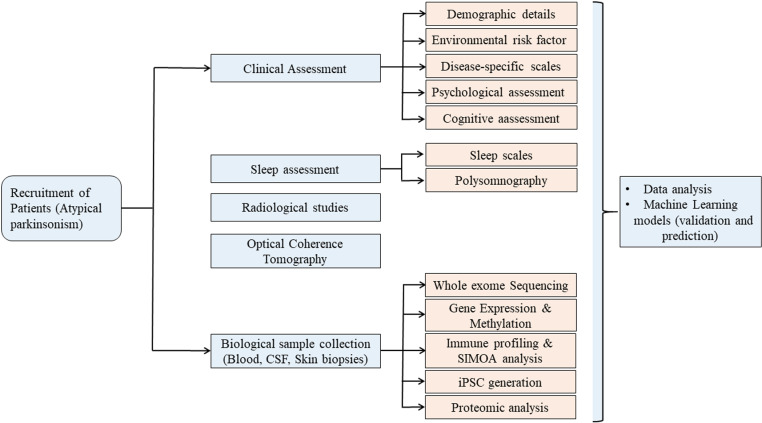
Comprehensive workflow for longitudinal analysis in atypical parkinsonism research protocol.

A longitudinal three-time point multicenter study has been adopted with a total sample size of 400 patients with APS. Patients will be recruited during the first three years of the study, anticipating a 10–12% of patient loss during follow-ups. The patients will be carefully selected after a detailed diagnosis by movement disorder specialists from the inpatient and outpatient services of the National Institute of Mental Health and Neurosciences (NIMHANS), Bangalore, India, and MS Ramaiah Medical College and Hospital, Bangalore, India. The socio-demographics detail and daily living habits such as age, gender, age of onset, economic class, place of living, profession, food habit, addiction, associated illness etc. will be obtained from the participants at the time of recruitment. The Movement Disorder Society (MDS) diagnostic criteria for PSP will be used to include and classify clinical variants of PSP [25]. Only probable and possible cases will be considered for the study. Participants with a diagnosis of clinically established MSA of the Parkinsonian subtype (MSA-P) or cerebellar subtype (MSA-C) will be included according to the MDS criteria for MSA [26]. The inclusion criteria for patients with CBS will be based on the Armstrong criteria [27]. The exclusion criteria for all the participants include being bedridden, having other neurodegenerative diseases, history of using sedatives, being pregnant/lactating women or no valid written informed consent. The comprehensive workflow of our ATPARK study is given in Fig 1.

### Ethical statement

The ethical approval for the study, including induced pluripotent stem cells (iPSC) culture was obtained from the Institutional Ethics Committee (NIMHANS/44^th^ IEC (BS&NS)/2023 dated 25^th^ September 2023). Moreover, ethical approvals for experiments involving human data at other centers will be obtained as required. A written informed consent will be obtained from each of the participants prior to the recruitment to the study.

### Follow-ups

For all participants, data will be collected at three time points, each one year apart. All the data including clinical, quality of life, cognitive functions, gene expressions, methylation patterns, inflammatory markers, disease-associated pathology markers, polysomnography (PSG), optical coherence tomography (OCT), and MRI will be conducted at each time point. Serial follow-ups at home visits will also be conducted in case of severe disease progression. Death during the study period will be recorded. The detailed plan for collecting the data at different time points is summarized in Table 1.

**Table 1:** The Study recruitment and follow-up plan and outline of the evaluation on each visit.

**Activities**	**Baseline**	**Follow-up** **(1**^**st**^ **year)**	**Follow-up** **(2**^**nd**^ **year)**
**Clinico-demographic data**			
** Demographic information**	**X**		
** Dietary & environmental risk factors**	**X**		
** Family history**	**X**		
** Clinical history**	**X**		
** Associated illness**	**X**		
**Neurological examination**			
** Disease-specific scales (PSPRS, UMSARS)**	**X**	**X**	**X**
** UPDRS-III**	**X**	**X**	**X**
**Cognitive assessment**			
** MOCA**	**X**	**X**	**X**
** FAB**	**X**	**X**	**X**
** ACE-III**	**X**	**X**	**X**
**Socio-psychological assessment**			
** R-CWBS**	**X**	**X**	**X**
** KCSS**	**X**	**X**	**X**
** MCSI**	**X**	**X**	**X**
** EACQ**	**X**	**X**	**X**
** NNBE**	**X**	**X**	**X**
** HAM-D & HAM-A**	**X**	**X**	**X**
**Sleep assessments**			
** Berlin questionnaire**	**X**	**X**	**X**
** Epworth sleepiness scale**	**X**	**X**	**X**
** Mayo sleep questionnaire**	**X**	**X**	**X**
** PSQI**	**X**	**X**	**X**
** RBD rating scale**	**X**	**X**	**X**
** RLS rating scale**	**X**	**X**	**X**
** PSG**	**X**	**X**	**X**
**Ocular function examination**			
** OCT**	**X**	**X**	**X**
**Brain imaging**			
** MRI**	**X**	**X**	**X**
**Bio-samples**			
** Whole exome sequencing**	**X**		
** Gene expression & epigenetic modification**	**X**	**X**	**X**
** Inflammation marker estimation**	**X**	**X**	**X**
** Non-targeted proteomics**	**X**	**X**	**X**
** Disease-associated biomarker analysis**	**X**	**X**	**X**

### Progress of the study

The study began in February 2024 and has completed over one year of recruitment. Data collection will continue until December 31, 2029, followed by data analysis.

### Clinical assessments

Motor and non-motor symptoms will be assessed using three key modules of clinical evaluations. Module 1 focuses on sleep assessment, including tools such as the Berlin Questionnaire for sleep apnea, the Epworth Sleepiness Scale (ESS) for daytime sleepiness, the Pittsburgh Sleep Quality Index (PSQI) for overall sleep quality over the past month, the Restless Legs Syndrome (RLS) Rating Scale for nighttime RLS discomfort, the Mayo Sleep Questionnaire and Rapid Eye Movement (REM) Sleep Behavior Disorder (RBD) Screening Questionnaire for sleep behavior disorder and dream-enactment behaviors. Module 2 assesses cognitive impairments using the Montreal Cognitive Assessment (MoCA), Frontal Assessment Battery (FAB), and Addenbrooke’s Cognitive Examination III (ACE-III). Module 3 evaluates disease severity using the Unified Parkinson’s Disease Rating Scale Part III (UPDRS-III), which will be applied to all groups, as well as the Progressive Supranuclear Palsy Rating Scale (PSPRS) for PSP and CBS cases, and the Unified Multiple System Atrophy Rating Scale (UMSARS) for MSA patients.

### Quality of life and neuropsychological assessments

These disorders are characterized by complex motor and non-motor symptoms, including severe manifestations, rapid progression, cognitive and behavioral issues, communication difficulties, financial strain, and emotional challenges, often requiring intensive caregiving. Socio-demographic data of caregivers will be collected using the Rapid Caregiver Well-being Scale (R-CWBS), Kingston Caregiver Stress Scale (KCSS), Modified Caregiver Strain Index (MCSI), and Experiential Avoidance in Caregiving Questionnaire (EACQ). Additionally, the neuropsychological status of the patients will be assessed using the NIMHANS Neuropsychological Battery for Elderly (NNBE), Kessler Psychological Distress Scale (K10), Hamilton Rating Scale for Depression (HAM-D), and Hamilton Rating Scale for Anxiety (HAM-A). Integrating these evaluations into the study will improve understanding of disease progression and its impact on both patients and caregivers.

### Biological samples

Approximately 16 ml of peripheral blood will be collected via venipuncture from the medial cubital vein of each participant, with 13 ml in ethylenediaminetetraacetic acid (EDTA) tubes and 3 ml in serum tubes. 5 ml of EDTA blood will be used for genomic DNA extraction, while the remainder will be used for plasma and peripheral blood mononuclear cells (PBMCs) isolation. Additionally, 3–4 ml of CSF will be collected from a subset of patients via lumbar puncture. All samples will be aliquoted into multiple tubes and stored at −80°C until analysis.

Moreover, iPSC lines will be generated from three patients in each experimental group, classified both clinically and genetically. Dopaminergic neurons and astrocytes will be differentiated from these iPSCs to study disease pathology. The expression of Tau4R and oligomeric α-synuclein, typically observed in classical PD-iPSC-derived dopaminergic neurons, will be analyzed [28].

Additionally, a punch skin biopsy will be performed under aseptic conditions and local anesthesia using a 3-mm disposable needle at the distal leg, 10 cm above the lateral malleolus in the sural nerve territory. Samples will be fixed in paraformaldehyde-lysine-periodate (PLP) fixative, cryoprotected in sucrose and sectioned (50–100 μm) perpendicular to the epidermis. Sections will be immunostained for phosphorylated α-synuclein at serine 129 (p-syn) and evaluated under a 10x objective for orientation, flatness, and staining quality. Staining with a pan-axonal marker (PGP9.5) will assess intra-epidermal nerve fibers (IENFs), with density calculated following European Federation of Neurological Societies and Peripheral Nerve Society guidelines [29].

### Genetic and epigenetic analysis

High-quality genomic DNA samples will be extracted from whole blood using the phenol-chloroform method and subjected to whole exome sequencing using Next Generation Sequencing (NGS) on a NovaSeq 6000 platform (Illumina, USA). The potential risk genes identified from sequencing data will be further analyzed for gene expression and promoter methylation studies. Gene expression will be quantified using an automated QX200 AutoDG Droplet Digital PCR System (Bio-Rad, USA) with TaqMan chemistry, while promoter methylation status will be assessed using a PyroMark Q48 Autoprep sequencer (Qiagen, Singapore).

### Immunological assessments

Th17 pathway-related cytokines will be estimated using a multiplex suspension array on the Bio-Plex 200 platform (Bio-Rad, Hercules, CA, USA) with the Bio-Plex Pro™ Human Th17 Cytokine Panel 15-Plex. This panel measures 15 cytokines associated with the Th17 pathway, including IL-6, IL-1β, IL-4, IL-10, IL-17A, IL-17F, IL-21, IL-22, IL-23, IL-25, IL-31, IL-33, IFN-γ, sCD40L, and TNF-α. All samples will be analyzed in duplicate to ensure accuracy and reliability.

### Proteomics and biomarker analysis

All CSF samples will undergo non-targeted proteomic analysis using liquid chromatography coupled with tandem mass spectrometry (LC-MS/MS). Protein detection and identification will be performed ona an Agilent Technologies 6545XT Advance Bio LC/Q-TOF equipped with Agilent 1290 Infinity II LC System. Data acquisition will be controlled by MassHunter workstation data acquisition software (B.08.01, Agilent) [30]. Additionally, the top three significantly upregulated proteins in the patient CSF will be further verified in CSF and serum using commercially available enzyme-linked immunosorbent assay (ELISA) kits and spectrophotometry (Tecan, Austria). Moreover, disease pathology-related candidate biomarkers including neurofilament light chain (NfL), α-synuclein, total Tau, p-Tau181, Amyloidβ-40, and Amyloidβ-42 will be estimated in plasma and CSF using Simoa platform in an automated HD-X analyzer (Quanterix, USA) following manufacturer’s instructions.

### Polysomnography (PSG) assessments

Whole-night PSG will be conducted following the American Academy of Sleep Medicine (AASM) guidelines [31] using a Somnomedics 33-channel PSG system (Randersacker, Germany). Standard recording channels will be used, including seventeen monopolar paste-based silver disc electrodes for the electroencephalogram (EEG), two electrodes for the electrooculogram (EOG), and two for the electromyogram (EMG). Additionally, actigraphy data will be recorded using a SOMNOwatch™ (SOW) system, which captures acceleration across three axes. The sleep parameters to be analyzed include total sleep time, wake duration, wake after sleep onset, sleep stages (N1, N2, N3, REM), their durations and latencies, arousal index, awakenings, sleep efficiency, maintenance, and REM episodes in each sleep stages.

### Optical Coherence Tomography (OCT)

OCT is a non-invasive method that reveals retinal changes, providing insights into the pathology of PD and atypical parkinsonism variants [24,32]. Eye movements will be assessed using automated eye tracking and video oculography. Visual acuity will be evaluated with Snellen’s chart, and retinal and optic nerve imaging will be performed using spectral-domain OCT (SD-OCT, Spectralis, Heidelberg Engineering, Heidelberg, Germany). Several imaging parameters such as Central macular thickness (CMT), Retinal nerve fiber layer thickness (RNFL), Subfoveal choroidal thickness (SFChT), Peri-papillary choroidal thickness (PPChT) will be considered for the analysis.

### Magnetic Resonance Imaging (MRI)

MRI imaging will be performed on a Philips 3T Ingenia scanner with multi-band sense technology, and using a 32-channel head coil. Several parameters will be considered for the MRI such as Anatomical Imaging, Diffusion Imaging, Advanced Blood-Oxygenation-Level-Dependent (BOLD) resting-state functional MRI with multi-band acquisitions, Arterial spin labelling and Substantia nigra imaging. A detailed methodology for the MRI data accuisation is given in the S1 Fig.

### Statistical analysis

The collected data will be entered into the Research Electronic Data Capture (REDCap) database [33] and appropriate descriptive statistics will be obtained for all study variables. The linearity of the data will be analyzed by Kolmogorov–Smirnov test. The clinical variables, gene copy numbers, epigenetic data, PSG parameters, OCT variables, MRI data, and biological markers will be compared for various socio-demographic variables within/across the groups using appropriate parametric or non-parametric univariate tests depending on the normality assumption and the type of variable. Survival analysis will be carried out to determine the factors associated with disease progression and also to estimate the risk of death. Survival time will be calculated from the date of enrolment for each participant to disease progression and death. Kaplan Meier plots will be used to compare the survival experience of various subgroups based on socio-demographic, clinical, and neurological parameters. A log-rank test will be used to test the statistical significance of the difference in survival distributions. The multivariable Cox proportional hazard model will be used to identify the prognostic factors of APS. Those variables with univariate P value < 0.05 will be considered statistically significant.

### Artificial intelligence/machine learning for multi-syndrome classification of APS

The proposed study on the longitudinal progression and the natural history of atypical Parkinsonism is expected to generate a large volume of high-dimensional datasets that include clinical markers, biochemical and genetic variables, and cognitive and neuroimaging data. Therefore, we aim to use principles of machine learning (ML) to evaluate the invariance, patterns, and representations in the data pertaining to clinical heterogeneity and survival, MRI morphology, genetic, and biochemical biomarkers. By applying unsupervised and supervised ML to deeply phenotyped patients, predictor models will be built to classify individual patients (S1 Fig). This would involve pre-processing steps to remove noise and artifacts, and to extract the relevant features. After feature selection, ML algorithms such as support vector machines (SVM), random forests, and neural networks (depending on data type), will be trained on the selected features to classify patients based on latent patterns of involvement into subtypes of APS. Models will be trained on 80% of the data and the remaining 20% of the data will be used for validation. The performance of the models will be evaluated using cross-validation and other established metrics, such as accuracy, sensitivity, specificity, and area under the receiver operating characteristic curve. The most informative features and biomarkers will be identified using feature importance and other interpretability techniques. Data analysis will be performed in Python using open-source softwares, and the code will be made publicly available.

## Discussion

The longitudinal studies in different continents, countries, and ethnic groups can reveal new insights about the risks, causality, epidemiological factors, survival, and care of patients with APS. One of the major published studies in this area is the PROSPECT-M-UK study, which recruited 243 patients, comprising 117 were PSP, 68 CBS, 42 MSA, and 16 were indeterminate [34]. The authors reported that phenotypic variability is the major challenge in patients with PSP, CBS and MSA. Additionally, they concluded that MRI was one of the most important tools for progression monitoring in these patients.

In another study, a multi-ethnic Asian PSP cohort of 104 patients (64.4% male; 67.3% Chinese, 21.2% Indians, 9.6% Malays), consisting of 48.1% Richardson syndrome (PSP-RS), 37.5% parkinsonian phenotype (PSP-P), and 10.6% progressive gait freezing phenotype (PSP-PGF) [35]. This study observed significant heterogeneity in clinical presentations and disease burden across different PSP phenotypes with high rates of RBD symptoms, and visual hallucinations. Another retrospective study from the USA and Canada analyzed 72 individuals with PSP, collecting an average of 144 medical documents per participant over a period of 7.9 years [36]. This study observed that the early onset of motor symptoms before diagnosis, the significant healthcare burden of PSP, and the widespread use of medical resources throughout the disease course.

Similarly, the Four Repeat Tauopathy Neuroimaging Initiative (4RTNI-2 Cycle 2) from the Memory and Aging Center in UCSD studied neuroimaging and CSF biomarkers in patients with 4R-tau disorders. They have identified several CSF proteins using proteomics in patients with PSP, specifically two inflammatory proteins galectin-10 and cytotoxic T lymphocyte-associated protein-4, which correlated with clinical PSP scores across cohorts [37]. They showed the axon guidance pathway protein abnormality in these patients.

Two of the National Institute of Health-supported studies, i) Advancing Research and Treatment in Frontotemporal Lobar Degeneration (ARTFL) (https://artfl-project.uchicago.edu/), and ii) Longitudinal Evaluation of Familial Frontotemporal Dementia Subjects (LEFFTDS) [38], have been merged into ARTFL–LEFFTDS Longitudinal Frontotemporal Lobar Degeneration (ALLFTD) research consortium. The main aim of this consortium was to better understand the Frontotemporal Lobar Degeneration (FTLD) syndromes and prepare the cohort for the clinical trials. This involved improving diagnostic accuracy using biomarker discovery and MRI neuroimaging, tascertaining the disease burden and finding ways to predict progression.

Indian patients with PSP possess unique genetic risk factors compared to other ethnicities, which highlights the need for population-specific studies to understand disease progression, severity, and potential therapeutic targets [39]. The current study aligns with previous longitudinal studies that tracked disease progression through clinical severity, pathological biomarkers in CSF, and neuroimaging matrices. Concurrently, our study will evaluate a variety of blood and CSF-based biomarkers comparatively in a larger underrepresented native cohort. Additionally, this study will explore the underlying pathogenetic mechanisms of disease onset and progression, considering the influence of genetic variations or their absence. Moreover, data analysis using ML will aid in identifying pathobiologically relevant blood-based biomarkers, which will not only support the diagnosis of APS but also facilitate clinical subtyping and the rate of progression across different diagnoses.

## Supporting information

S1 Fig The pipeline for developing and deploying machine learning models for multi-syndrome classification of atypical parkinsonism. (TIF)
