# Supplementary figures and images for: Progression and natural history of Atypical Parkinsonism (ATPARK): Protocol for a longitudinal follow-up study from an underrepresented population
Source: PLoS One. 2025 Jun 26;20(6):e0325624. doi: 10.1371/journal.pone.0325624 (PMC12200670; doi:10.1371/journal.pone.0325624)

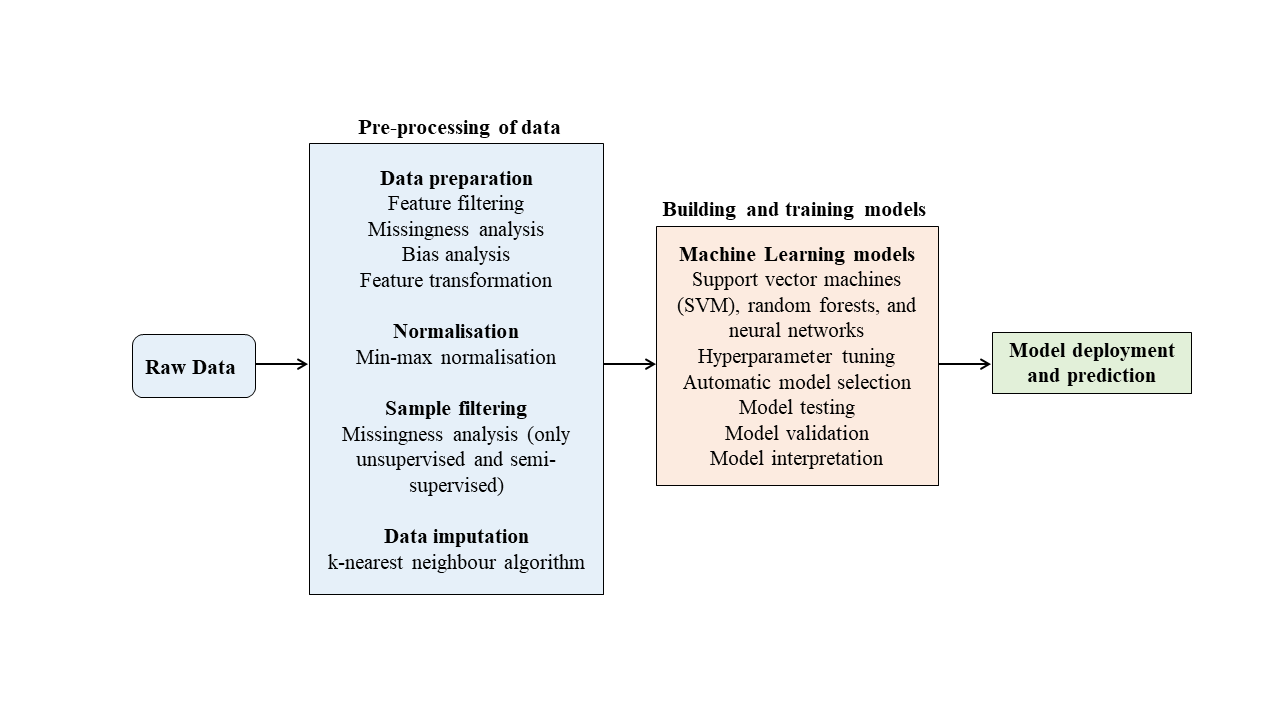

Supplement: S1 Fig — (TIF) [file pone.0325624.s001.tif]
